# Supplementary material for: Rapid and simultaneous detection of Campylobacter spp. and Salmonella spp. in chicken samples by duplex loop-mediated isothermal amplification coupled with a lateral flow biosensor assay
Source: PLoS One. 2021 Jul 1;16(7):e0254029. doi: 10.1371/journal.pone.0254029 (PMC8248736; doi:10.1371/journal.pone.0254029)
Supplement: S1 Fig — C and T indicate the control and test lines, respectively. A positive result displayed bands at both C and T, while a negative result showed one band at the C line. 1: S. Enteritidis DMST 17368; 2: S. Enteritidis DMST 33954; 3: S. Typhi DMST 22842; 4: S. Typhimurium DMST 2069; 5: S. Typhimurium DMST 16150; 6: S. Typhimurium DMST 16152; 7–36: Campylobacter spp. isolates (M-23, 26, 45, 51, 62, 91, 93, 97, 152, 165, 198, 213, 272, 351, 352, 363, 370, 379, 392, 399, 406, 413, 417, 430, 441, 445, 449, 450, 451 and 464); 37: S. Choleraesuis; 38: S. Hadar; 39: S. Infantis; 40: S. Paratyphi A; 41: S. Virchow. (PDF) [file pone.0254029.s001.pdf]

S1 Fig.

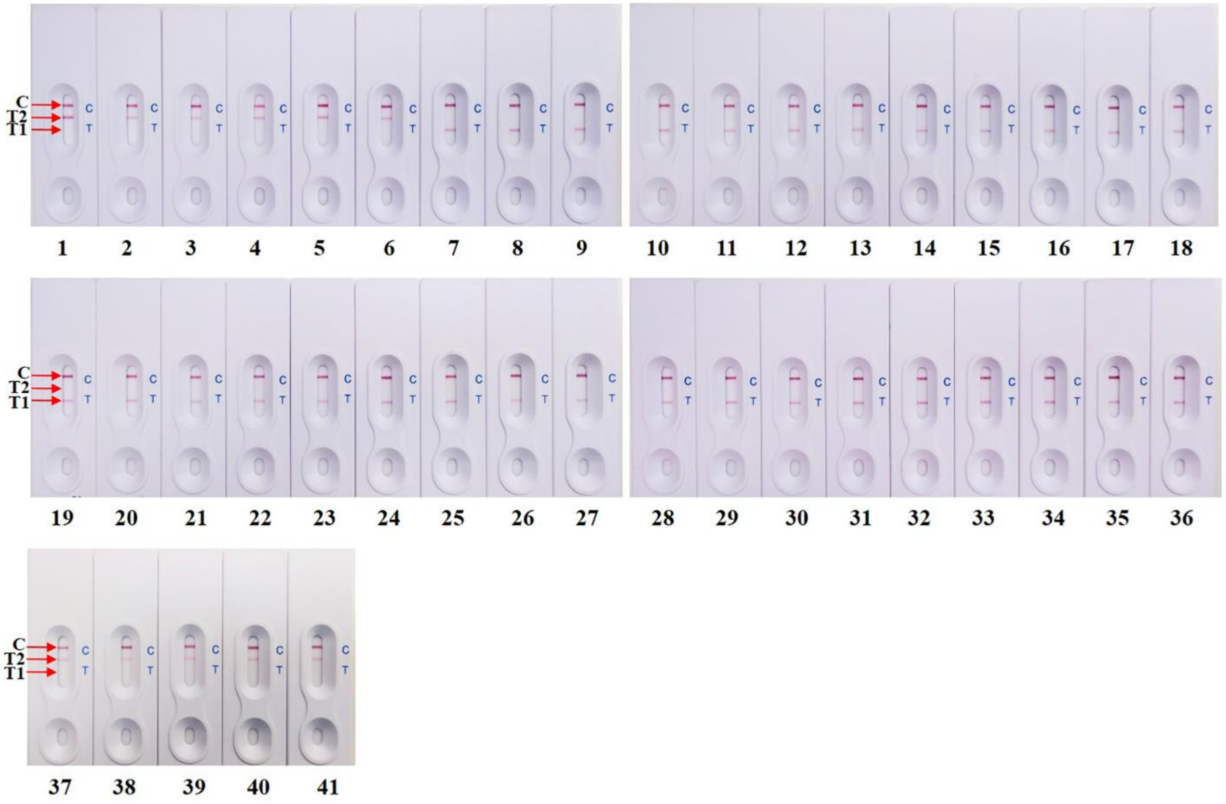

S1 Fig. The specificity of d-LAMP-LFB assay for detecting different strains of *Campylobacter and Salmonella* spp. using 20 ng each of DNA templates. C and T indicate the control and test lines, respectively. A positive result displayed bands at both C and T, while a negative result showed one band at the C line. 1: *S*. Enteritidis DMST 17368; 2: *S*. Enteritidis DMST 33954; 3: *S*. Typhi DMST 22842; 4: *S*. Typhimurium DMST 2069; 5: *S*. Typhimurium DMST 16150; 6: *S*. Typhimurium DMST 16152; 7-36: *Campylobacter* spp. isolates (M-23, 26, 45, 51, 62, 91, 93, 97, 152, 165, 198, 213, 272, 351, 352, 363, 370, 379, 392, 399, 406, 413, 417, 430, 441, 445, 449, 450, 451 and 464); 37: *S*. Choleraesuis; 38: *S*. Hadar; 39: *S*. Infantis; 40: *S*. Paratyphi A; 41: *S*. Virchow.
