# Supplementary material for: Rapid and simultaneous detection of Campylobacter spp. and Salmonella spp. in chicken samples by duplex loop-mediated isothermal amplification coupled with a lateral flow biosensor assay
Source: PLoS One. 2021 Jul 1;16(7):e0254029. doi: 10.1371/journal.pone.0254029 (PMC8248736; doi:10.1371/journal.pone.0254029)
Supplement: S2 Fig — A: UFUL and URUL primer; B: Sal1598 F and Sal1859 R primer; Sample 1–30 obtained from the retail markets; Lane M: 2-log DNA ladder; Lane P: positive control (S. Typhimurium ATCC 23566); Lane N: blank control (the reaction with 2 μl sterile distilled water). (PDF) [file pone.0254029.s002.pdf]

S2 Fig.

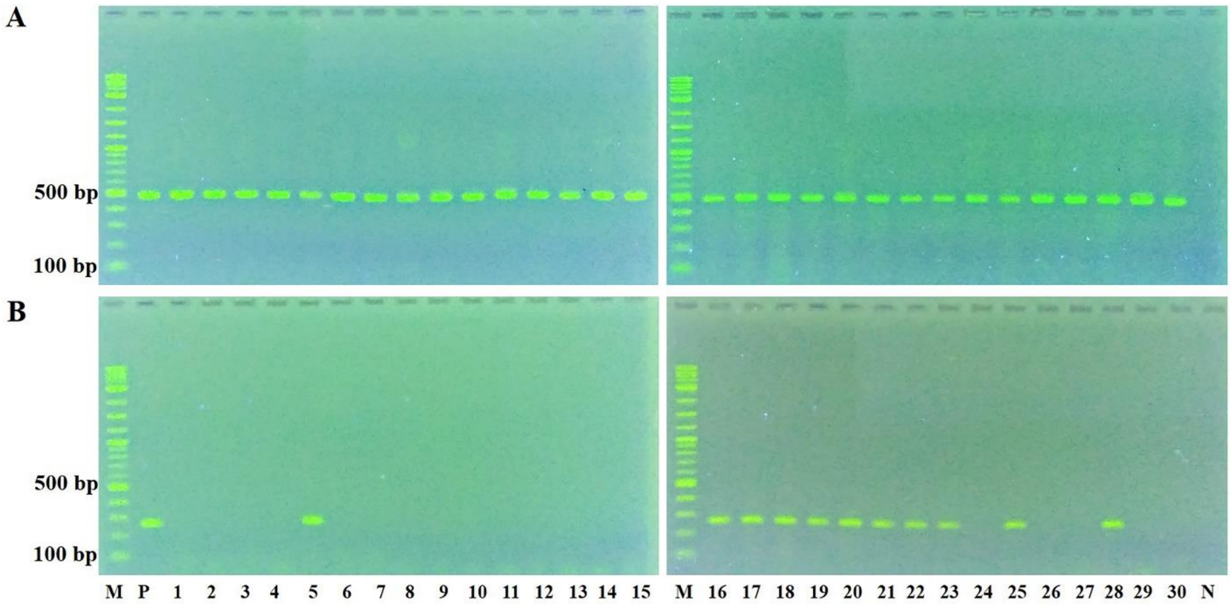

S2 Fig. Agarose gel electrophoresis of PCR products obtained from the DNA extracted from colonies in RV medium based-XLD agar for the detecting *Salmonella* spp. by culture based-method. A: UFUL and URUL primer; B: Sal1598 F and Sal1859 R primer; Sample 1-30 obtained from the retail markets; Lane M: 2-log DNA ladder; Lane P: positive control (*S*. Typhimurium ATCC 23566); Lane N: blank control (the reaction with 2 µl sterile distilled water).
