# Supplementary material for: Rapid and simultaneous detection of Campylobacter spp. and Salmonella spp. in chicken samples by duplex loop-mediated isothermal amplification coupled with a lateral flow biosensor assay
Source: PLoS One. 2021 Jul 1;16(7):e0254029. doi: 10.1371/journal.pone.0254029 (PMC8248736; doi:10.1371/journal.pone.0254029)
Supplement: S3 Fig — A: UFUL and URUL primer; B: Sal1598 and Sal1859 R primer; Sample 1–30 obtained from the retail markets; Lane P: positive control (S. Typhimurium ATCC 23566); Lane N: blank control (the reaction with 2 μl sterile distilled water). (PDF) [file pone.0254029.s003.pdf]

**S3 Fig.**

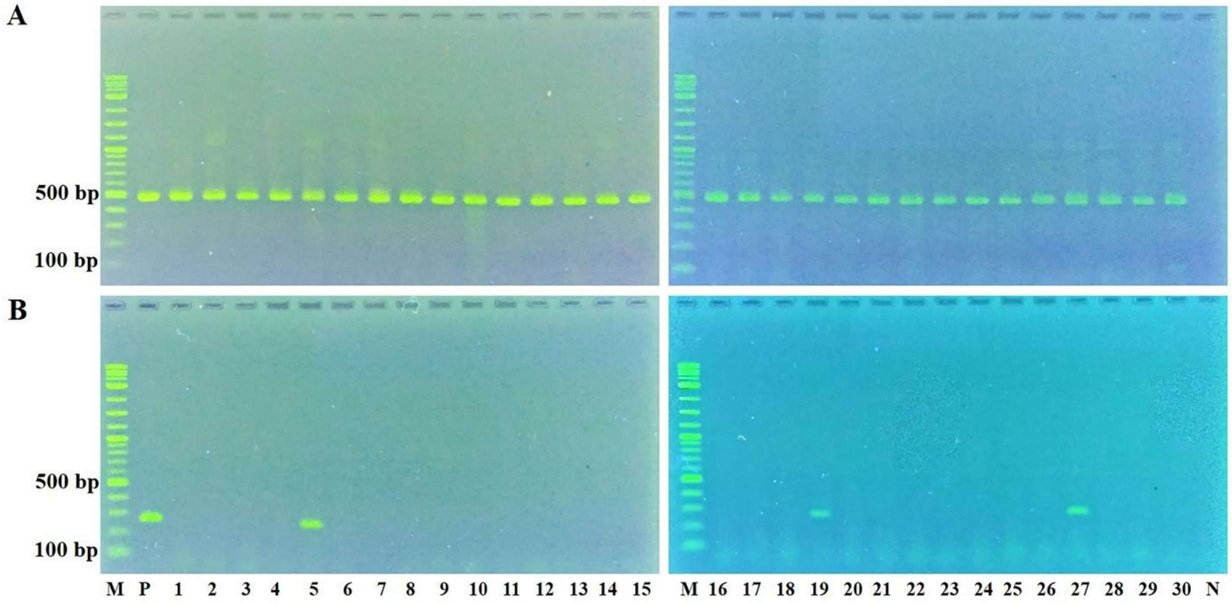


**S3 Fig. Agarose gel electrophoresis of PCR products obtained from the DNA extracted from colonies in TT medium based- XLD agar for the detecting *Salmonella* spp. by culture based-method.** A: UFUL and URUL primer; B: Sal1598 and Sal1859 R primer; Lane 1-30 obtained from the retail markets; Lane P: positive control (*S*. Typhimurium ATCC 23566); Lane N: blank control (the reaction with 2 µl sterile distilled water).
