# Supplementary material for: Rapid and simultaneous detection of Campylobacter spp. and Salmonella spp. in chicken samples by duplex loop-mediated isothermal amplification coupled with a lateral flow biosensor assay
Source: PLoS One. 2021 Jul 1;16(7):e0254029. doi: 10.1371/journal.pone.0254029 (PMC8248736; doi:10.1371/journal.pone.0254029)
Supplement: S4 Fig — A: UFUL and URUL primer; B: 16S-F and 16S-R primer; Sample 7–30 obtained from the retail markets; Lane P: positive control (C. jejuni DMST 15190); Lane N: blank control (the reaction with 2 μl sterile distilled water). (PDF) [file pone.0254029.s004.pdf]

S4 Fig.

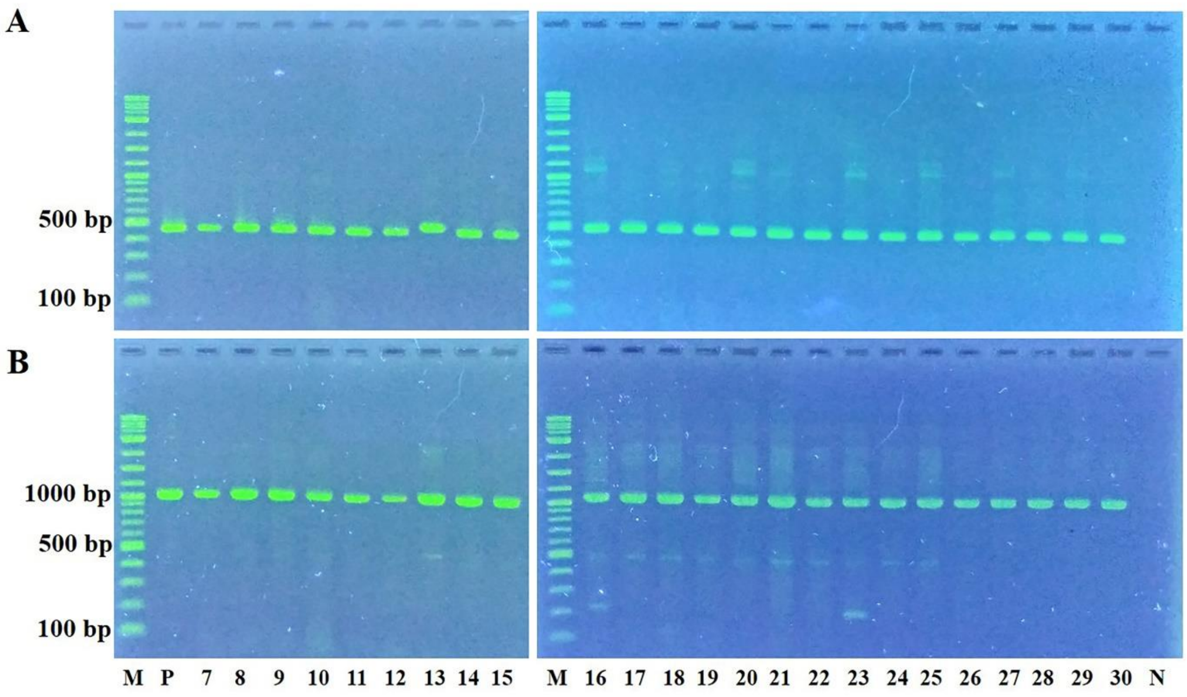

S4 Fig. Agarose gel electrophoresis of PCR products obtained from the DNA extracted from colonies in Bolton agar for the detecting *Campylobacter* spp. by culture based-method. A: UFUL and URUL primer; B: 16S-F and 16S-R primer; Sample 7-30 obtained from the retail markets; Lane P: positive control (*C. jejuni* DMST 15190); Lane N: blank control (the reaction with 2 µl sterile distilled water).
