# Supplementary material for: Rapid and simultaneous detection of Campylobacter spp. and Salmonella spp. in chicken samples by duplex loop-mediated isothermal amplification coupled with a lateral flow biosensor assay
Source: PLoS One. 2021 Jul 1;16(7):e0254029. doi: 10.1371/journal.pone.0254029 (PMC8248736; doi:10.1371/journal.pone.0254029)
Supplement: S1 Table — (PDF) [file pone.0254029.s005.pdf]

S1 Table. Bacterial strains used in this study to determine the specificity of the designed primers for d-LAMP-LFB assay.

Bacterial species	Serogroup	Serovar	Strains	d-LAMP-LFB results	
				T1 line	T2 line
Salmonella enterica	B	Agona	DMST 10638	-	+
Salmonella enterica	B	Abony	DMST 21863	-	+
Salmonella enterica	E1	Anatum	DMST 16870	-	+
Salmonella enterica	Z	Arizonae	DMST 22439	-	+
Salmonella enterica	P	Bangkok	DMST 7121	-	+
Salmonella enterica	X	Bergen	DMST 10895	-	+
Salmonella enterica	K	Cerro	DMST 17381	-	+
Salmonella enterica	C1	Choleraesuis	Laboratory	-	+
Salmonella enterica	B	Derby	DMST 8535	-	+
Salmonella enterica	D	Enteritidis	DMST 15676	-	+
Salmonella enterica	D	Enteritidis	DMST 17368	-	+
Salmonella enterica	D	Enteritidis	DMST 33954	-	+
Salmonella enterica	D	Gallinarum	DMST 15968	-	+
Salmonella enterica	C2	Hadar	Laboratory	-	+
Salmonella enterica	I	Hvittingfoss	DMST 15681	-	+
Salmonella enterica	C1	Infantis	Laboratory	-	+
Salmonella enterica	C1	Mbandaka	DMST 17377	-	+
Salmonella enterica	C2	Newport	DMST 15675	-	+
Salmonella enterica	D	Panama	DMST 10640	-	+
Salmonella enterica	A	Paratyphi A	Laboratory	-	+
Salmonella enterica	B	Paratyphi B	DMST 28118	-	+
Salmonella enterica	G	Poona	DMST 15679	-	+
Salmonella enterica	B	Schwarzengrund	DMST 17364	-	+
Salmonella enterica	E4	Senftenberg	DMST 17013	-	+
Salmonella enterica	B	Stanley	DMST 16874	-	+
Salmonella enterica	D	Typhi	DMST 5784	-	+
Salmonella enterica	D	Typhi	DMST 22842	-	+
Salmonella enterica	B	Typhimurium	ATCC 23566	-	+
Salmonella enterica	B	Typhimurium	DMST 562	-	+

S1 Table. (continued)

Bacterial species	Serogroup	Serovar	Strains	d-LAMP-LFB results	
				T1 line	T2 line
Salmonella enterica	B	Typhimurium	DMST 2069	-	+
Salmonella enterica	B	Typhimurium	DMST 16150	-	+
Salmonella enterica	B	Typhimurium	DMST 16152	-	+
Salmonella enterica	C1	Virchow	Laboratory	-	+
Salmonella enterica	Q	Wandsworth	DMST 19204	-	+
Salmonella enterica	S	Waycross	DMST 19205	-	+
Campylobacter jejuni			DMST 15190	+	-
Campylobacter coli			DMST 18034	+	-
Campylobacter lari			DMST 17953	+	-
Campylobacter spp.			M-23	+	-
Campylobacter spp.			M-26	+	-
Campylobacter spp.			M-45	+	-
Campylobacter spp.			M-51	+	-
Campylobacter spp.			M-62	+	-
Campylobacter spp.			M-91	+	-
Campylobacter spp.			M-93	+	-
Campylobacter spp.			M-97	+	-
Campylobacter spp.			M-152	+	-
Campylobacter spp.			M 165	+	-
Campylobacter spp.			M-198	+	-
Campylobacter spp.			M-213	+	-
Campylobacter spp.			M-272	+	-
Campylobacter spp.			M-351	+	-
Campylobacter spp.			M-352	+	-
Campylobacter spp.			M-363	+	-
Campylobacter spp.			M-370	+	-
Campylobacter spp.			M 379	+	-
Campylobacter spp.			M-392	+	-
Campylobacter spp.			M-399	+	-
Campylobacter spp.			M-406	+	-

S1 Table. (continued)

Bacterial species	Serogroup	Serovar	Strains	d-LAMP-LFB results	
				T1 line	T2 line
Campylobacter spp.			M-413	+	-
Campylobacter spp.			M-417	+	-
Campylobacter spp.			M-430	+	-
Campylobacter spp.			M-441	+	-
Campylobacter spp.			M-445	+	-
Campylobacter spp.			M-449	+	-
Campylobacter spp.			M-450	+	-
Campylobacter spp.			M-451	+	-
Campylobacter spp.			M-464	+	-
Bacillus cereus			ATCC 14579	-	-
Enterobacter aerogenes			DMST 2720	-	-
Escherichia coli			DMST 703	-	-
Escherichia coli			DMST 4212	-	-
Listeria monocytogenes			DMST 17303	-	-
Shigella boydii			DMST 30245	-	-
Staphylococcus aureus			ATCC 25923	-	-
Staphylococcus epidermidis			DMST 15505	-	-
Staphylococcus haemolyticus			DMST 15511	-	-
Vibrio cholera			DMST 2873	-	-
Vibrio vulnificus			DMST 21245	-	-
Yersinia enterocolitica			DMST 8012	-	-

ATCC: American Type Culture Collection, USA.

DMST: Department of Medical Sciences Thailand, Thailand

Laboratory: Department of Biology, Faculty of Science, Mahidol University, Bangkok, Thailand

M: *Campylobacter* spp. isolate strains

(“+”, The assay is visualized on T1 or T2 line by naked eye; “-”, The assay is not visualized on T1 or T2 line; C line always visible in all test)
