# Supplementary material for: Rapid and simultaneous detection of Campylobacter spp. and Salmonella spp. in chicken samples by duplex loop-mediated isothermal amplification coupled with a lateral flow biosensor assay
Source: PLoS One. 2021 Jul 1;16(7):e0254029. doi: 10.1371/journal.pone.0254029 (PMC8248736; doi:10.1371/journal.pone.0254029)
Supplement: S2 Table — (PDF) [file pone.0254029.s006.pdf]

S2 Table. Primer sequences and conditions used for the PCR assay.

Species	Primer	Sequence (5'-3')	Target gene	Size (bp)	Reference
Bacterial species	UFUL	GCCTAACACATGCAAGTCGA	16S rRNA	492	Nilsson et al., 2002
	URUL	CGTATTACCGCGGCTGCTGG			
Campylobacter spp.	16S-F	GGAGGCAGCAGTAGGGAATA	16S rRNA	1,062	Persson et al., 2005
	16S-R	TGACGGGCGGTGAGTACAAG			
Salmonella spp.	Sal1598 F	AACGTGTTTCCGTGCGTAAT	*invA*	262	Cheng et al., 2008
	Sal1859 R	TCCATCAAATTAGCGGAGGC			

PCR condition

PCR amplification was carried out in a 20 μl mixture containing 1×supplied buffer, 1.5 mmol l^{-1} $MgCl_2$, (Vivantis, Malaysia), 0.2 mmol l^{-1} dNTPs (Vivantis, Malaysia), 1 U of *Taq* DNA polymerase (Vivantis, Malaysia), 0.2 μmol l^{-1} each of forward and reverse primer (Biobasic, Canada) and 2 μl of DNA template. The reaction was performed in a thermal cycler (Biorad, USA) with the following program: initial denaturation at 94 °C for 2 min, 30 cycles each consisting denaturation at 94 °C for 30 s, annealing at 55 °C for 30 s, extension at 72 °C for 30 s and a final extension step at 72 °C for 7 min. 2 μl of PCR products were verified using 1.5% agarose gel electrophoresis, followed by staining with Serva DNA stain G (SERVA Electrophoresis GmbH, Germany). The gel image was taken under UV light using the mini UV table ultraviolet analyzer (Extragene, Taiwan).
