# Supplementary material for: Rapid and simultaneous detection of Campylobacter spp. and Salmonella spp. in chicken samples by duplex loop-mediated isothermal amplification coupled with a lateral flow biosensor assay
Source: PLoS One. 2021 Jul 1;16(7):e0254029. doi: 10.1371/journal.pone.0254029 (PMC8248736; doi:10.1371/journal.pone.0254029)
Supplement: S3 Table — (PDF) [file pone.0254029.s007.pdf]

S3 Table. Comparison of d-LAMP-LFB assay results and culture-based method for simultaneous detection of *Campylobacter* and *Salmonella* spp. in raw chicken meat samples.

d-LAMP-LFB	Culture-based method		Total	Sensitivity (%) (95% CI)	Specificity (%) (95% CI)	Accuracy (%) (95% CI)
	Positive	Negative				
Positive	22	2	24	95.6 (78.0-99.8)	71.4 (29.0-96.3)	90.0 (73.4-97.8)
Negative	1	5	6			
Total	23	7	30			
