# Supplementary material for: Rapid and simultaneous detection of Campylobacter spp. and Salmonella spp. in chicken samples by duplex loop-mediated isothermal amplification coupled with a lateral flow biosensor assay
Source: PLoS One. 2021 Jul 1;16(7):e0254029. doi: 10.1371/journal.pone.0254029 (PMC8248736; doi:10.1371/journal.pone.0254029)
Supplement: S1 Raw images — (PDF) [file pone.0254029.s009.pdf]

Original photographs of Fig 2A.

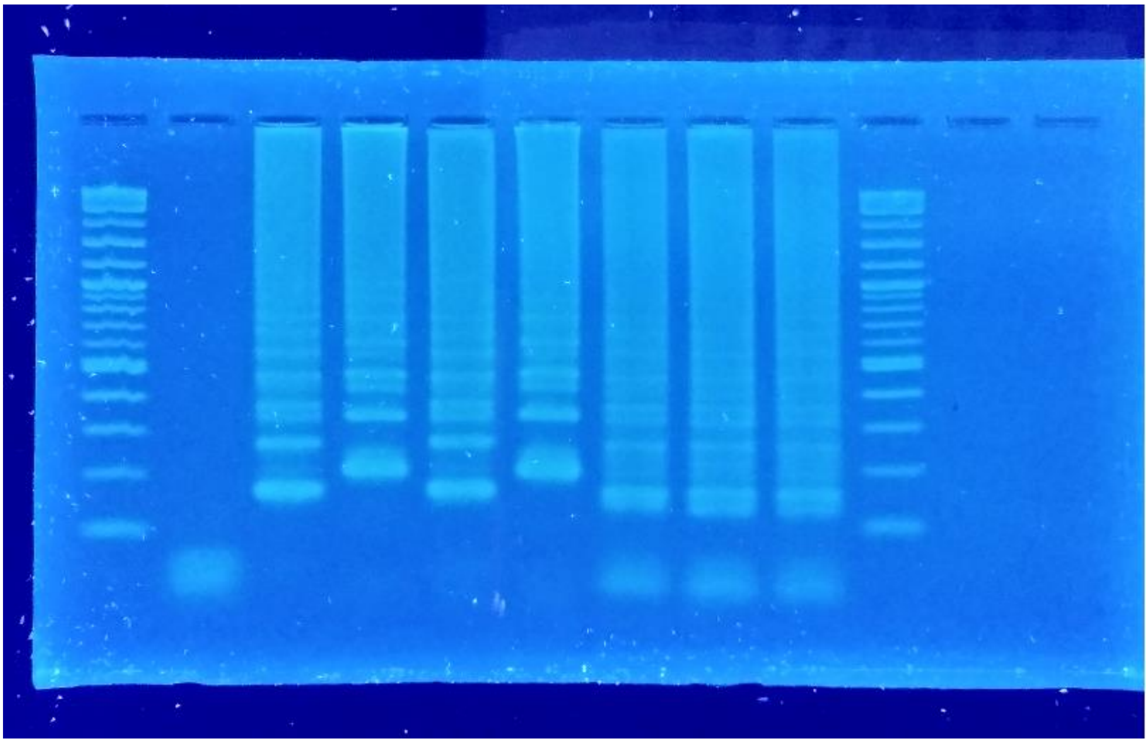

M Blank S C S C S+C S+C S+C M

s-LAMP-LFB d-LAMP-LFB

M: The DNA marker is 2-log DNA ladder (0.1-10.0 kb, No. N3200S) (New England Biolabs Inc., USA); S: LAMP product of *S*. Typhimurium; C: LAMP product of *C. jejuni*; S+C: LAMP product of *S*. Typhimurium+*C. jejuni*; The photo was taken with the mini UV table ultraviolet analyzer (Extragene, Taiwan).

Original photographs of S2 Fig.

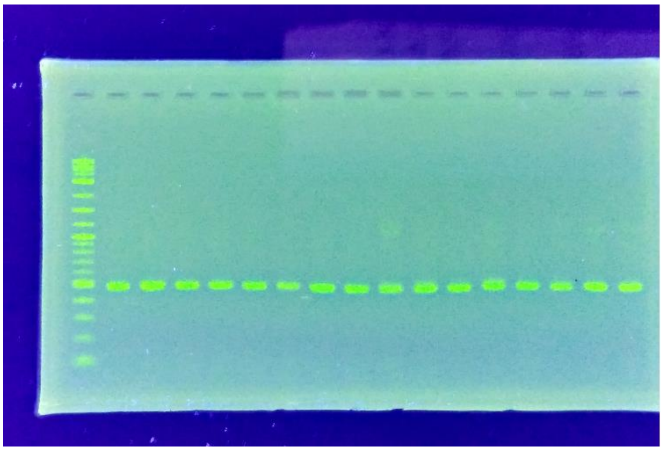

M P 1 2 3 4 5 6 7 8 9 10 11 12 13 14 15

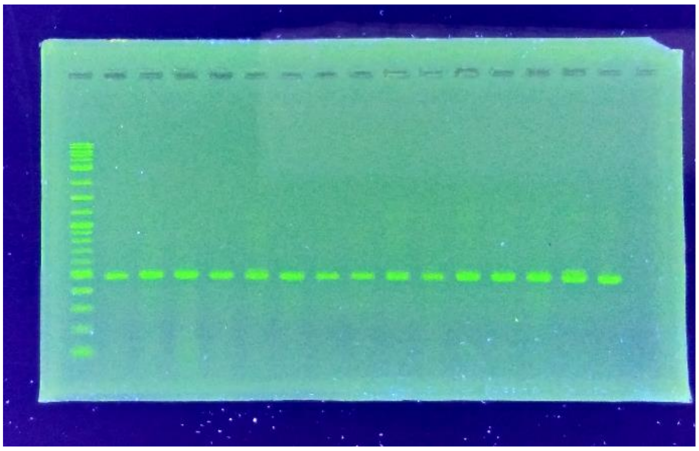

M 16 17 18 19 20 21 22 23 24 25 26 27 28 29 30 N

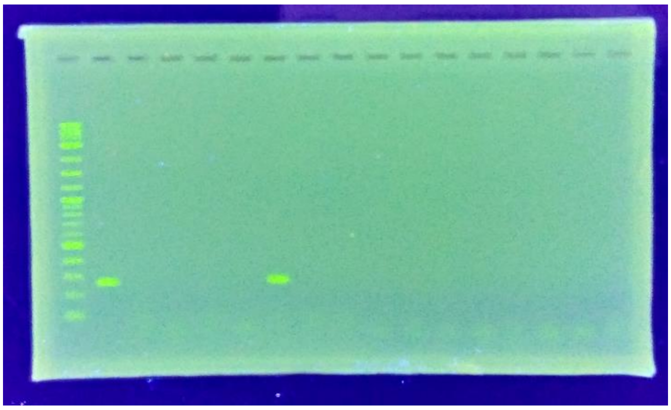

M P 1 2 3 4 5 6 7 8 9 10 11 12 13 14 15

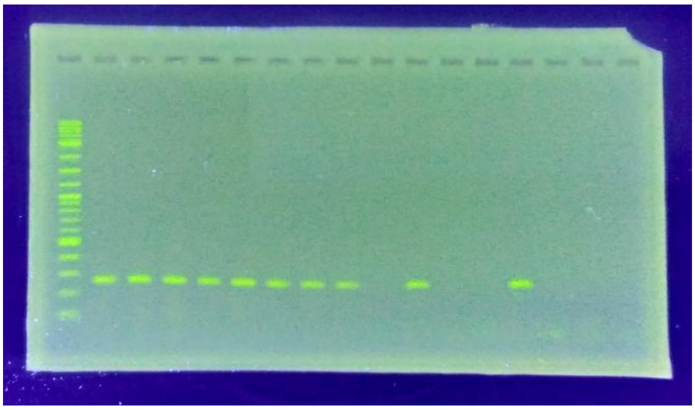

M 16 17 18 19 20 21 22 23 24 25 26 27 28 29 30 N

The DNA marker is 2-log DNA ladder (0.1-10.0 kb, No. N3200S) (New England Biolabs Inc., USA). The photo was taken with the mini UV table ultraviolet analyzer (Extragene, Taiwan).

Original photographs of S3 Fig.

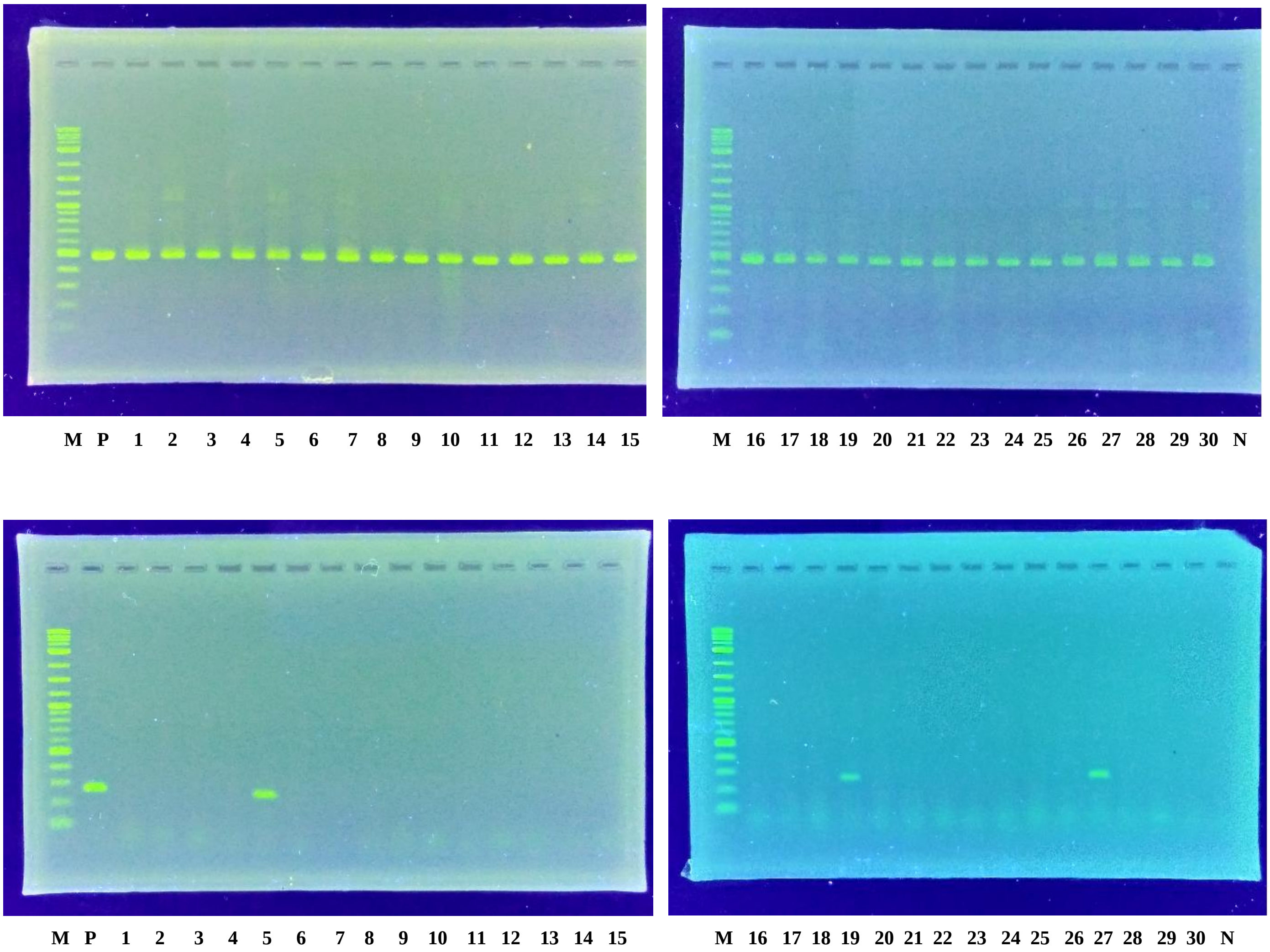

The DNA marker is 2-log DNA ladder (0.1-10.0 kb, No. N3200S) (New England Biolabs Inc., USA). The photo was taken with the mini UV table ultraviolet analyzer (Extragene, Taiwan).

Original photographs of S4 Fig.

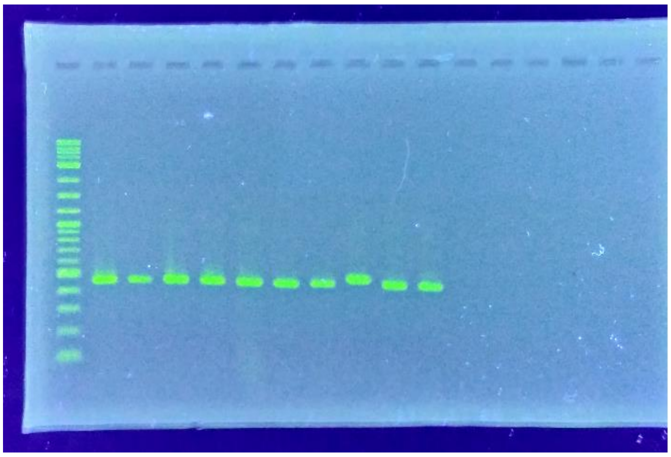

M P 7 8 9 10 11 12 13 14 15

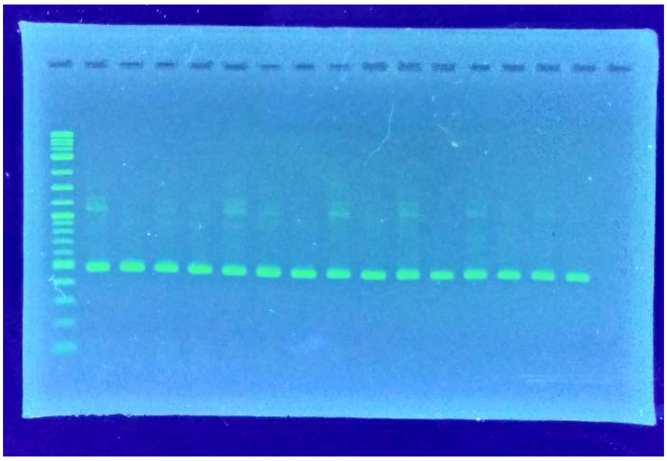

M 16 17 18 19 20 21 22 23 24 25 26 27 28 29 30 N

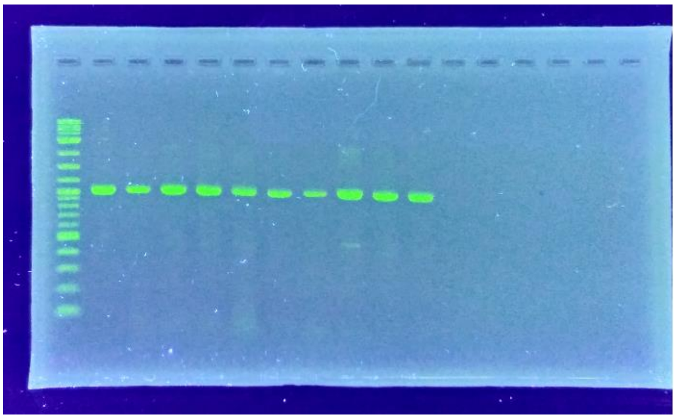

M P 7 8 9 10 11 12 13 14 15

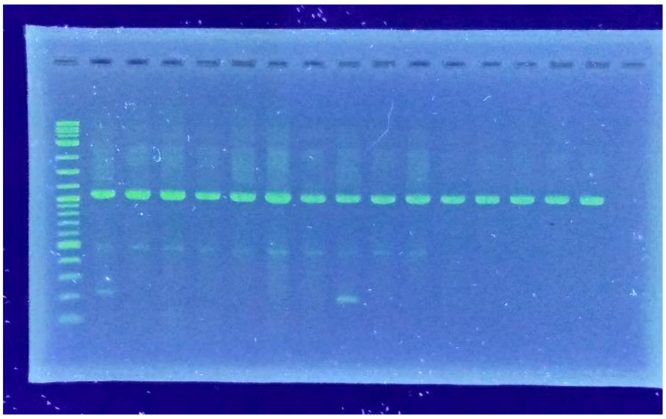

M 16 17 18 19 20 21 22 23 24 25 26 27 28 29 30 N

The DNA marker is 2-log DNA ladder (0.1-10.0 kb, No. N3200S) (New England Biolabs Inc., USA). The photo was taken with the mini UV table ultraviolet analyzer (Extragene, Taiwan).
