# Supplementary material for: Rapid and simultaneous detection of Campylobacter spp. and Salmonella spp. in chicken samples by duplex loop-mediated isothermal amplification coupled with a lateral flow biosensor assay
Source: PLoS One. 2021 Jul 1;16(7):e0254029. doi: 10.1371/journal.pone.0254029 (PMC8248736; doi:10.1371/journal.pone.0254029)
Supplement: Attachment — Submitted filename: Response to the reviewer 280421 final.pdf [file pone.0254029.s011.pdf]

Response to the reviewer' comments

Page number indicated below refers to that of the "Revised Manuscript with Track Changes" unless stated.

The modified text was marked in Track changes.

Journal Requirements#:

1: Please ensure that your manuscript meets PLOS ONE's style requirements, including those for file naming. The PLOS ONE style templates can be found at https://journals.plos.org/plosone/s/file?id=wjVg/PLOSOne_formatting_sample_main_body.pdf and https://journals.plos.org/plosone/s/file?id=ba62/PLOSOne_formatting_sample_title_authors_affiliations.pdf

***Response*:** The revised manuscript meets PLOS ONE's style requirements.

2: PLOS ONE now requires that authors provide the original uncropped and unadjusted images underlying all blot or gel results reported in a submission's figures or Supporting Information files. This policy and the journal's other requirements for blot/gel reporting and figure preparation are described in detail at https://journals.plos.org/plosone/s/figures#loc-blot-and-gel-reporting-requirements and https://journals.plos.org/plosone/s/figures#loc-preparing-figures-from-image-files. When you submit your revised manuscript, please ensure that your figures adhere fully to these guidelines and provide the original underlying images for all blot or gel data reported in your submission. See the following link for instructions on providing the original image data: https://journals.plos.org/plosone/s/figures#loc-original-images-for-blots-and-gels.

***Response*:** Original images for all gel data reported in the revised manuscript are provided in Supporting Information file (PDF file).

Reviewer #1: General comments:

The manuscript described a d-LAMP-LFB assay for rapid, sensitive and simultaneous detection of *Campylobacter* spp. and *Salmonella* spp.in the chicken samples in one assay. In my opinion, the assay is technically sound, and the experimental data support the conclusion. There is only one minor question needed to emphasize.

Response**:** We thank the reviewer#1 for careful reading of our manuscript. We are grateful for your consideration of this manuscript.

Reviewer #1: Specific comments:

Introduction:

1. Line 66; previous version: You mentioned that the real-time PCR kits for food-borne pathogens are costly. According to my knowledge, the real-time PCR is less expensive than the LAMP, especially the LAMP combined with lateral flow biosensor.

Response**:** We appreciate your comment. Till date, real-time PCR kits are commercially available for detecting foodborne pathogens (1), and the cost of kits is slightly less expensive than that of developed LAMP-LFB. However, most real-time detection kits can detect only a single pathogen either *Campylobacter* or *Salmonella* species. The time and reagent cost for independently detecting these pathogens were increased. Meanwhile, d-LAMP-LFB in this study can detect both pathogens simultaneously, which was more rapid, time-saving and effort without compromising the results. The reagent cost for d-LAMP-LFB assay was reduced and found to be cheaper than that of real-time PCR kits. In addition, real-time PCR kits required real-time PCR machine, which is highly sophisticated instrument to operate. Real-time PCR technique can be used in well-equipped laboratories that is unsuitable for use in field, point-of-care and resource-limited settings (2). Meanwhile, the LAMP technique is based on auto-cycling strand displacement DNA synthesis carried out by *Bst* DNA polymerase at a single temperature in the range of 60-65 °C to amplified nucleic acid in a single step, eliminating the use of a costly specialized instrument. Only heating block or simple water baths, which are common and cost-effective reaction equipment can be used (1, 3, 4). The advantage of low-cost and easy operation of LAMP could be adapted for use in a lack of infrastructure to support PCR facilities. In conclusion, the ability to simultaneously analyze multiple targets in a single tube can translate to increased time and cost-efficiency, yielding more

data from each reaction and utilization of fewer reagents. This information is included in our revised manuscript on **Page 3, Line 66-67.**

Reviewer #2: General comments:

1. The authors indicated that the d-LAMP is rapid and simple for simultaneous detection of *Salmonella* spp. and *Campylobacter* spp. However, this technique must rely on pre-enrichment which is the bottle neck of the overall process and make it not rapid and simple as claimed by the authors. Though the pre-enrichment has the major advantage in discrimination between dead and alive pathogenic bacteria, but it is tedious and time- consuming. Hence, the overall process starting from sample treatment could take approximately 24 hours or more which is not rapid and simple. As such, line 398-391 should be deleted or re-discussed since the d-LAMP combined with pre-enrichment could not be applied for field investigation.

***Response*:** We appreciate your comment which helped improving the quality of the paper. We answer the reviewer's concerns as:

a) This technique must rely on pre-enrichment.

***Response*:** To date, several molecular detection methods such as PCR, qPCR and LAMP assays have been developed for detection of pathogens from foods (1, 3). However, most are unable to detect positive samples without pre-enrichment step (5, 6). The sufficient limit of detection (LOD) of the test is important to reliably detect pathogens in foods according to the legal requirements, to reduce the chance of false negatives due to low numbers of *Campylobacter* and *Salmonella* cells in samples with high background microflora (7). Thus, an enrichment step is still necessary prior to those molecular assays (8-13). An enrichment step serves purposes as: (i) to increase bacterial cell numbers into the detection range, (ii) to discriminate between live and dead cells and (iii) to dilute interfering substances such as organic and inorganic chemicals, polysaccharides present in the sample matrix (6, 14). This information is included in our revised manuscript.

b) Overall process starting from sample treatment could take approximately 24 hours or more which is not rapid and simple.

***Response*:** We agree with the reviewer's comment. Our developed d-LAMP-LFB assay coupled with enrichment culture procedure could take approximately 24 hours or more. However, our d-LAMP-LFB assay provides a result as soon as next day after sample receipt within two 8-h working shifts (including 24 h of enrichment, DNA extraction and d-LAMP-LFB detection) and

requires less labor and equipment. Unlike gold standard culture methods that require over 5-7 workdays for sampling, culture, enumeration, and biochemical confirmation of the presumptive bacterial colonies according to the ISO10272:2006 (*Campylobacter* spp.) and ISO6579:2002 (*Salmonella* spp.) guidelines (15, 16) are time consuming, required various instruments and labor intensive. The advantage of d-LAMP-LFB can also detect both pathogens simultaneously, which offers a more rapid detection compared to daily procedures on each standard culture method. Thus, developed d-LAMP-LFB in this study could serve as an alternative to the gold standard culture method. This information is included in our revised manuscript.

c) Line 389-391; previous version; should be deleted or re-discussed since the d-LAMP combined with pre-enrichment could not be applied for field investigation.

***Response*:** Thank you for this remark. We apologize for such confusion. The phrase "*field food samples*" means "*real food samples at the retail markets*" to describe that our developed d-LAMP-LFB assay offers a valuable detection tool for its field applicability by detecting of both *Campylobacter* and *Salmonella* present in real food samples. As the reviewer's suggestion, the word "*field*" has been removed on **Page 18, Line 392** to avoid confusion.

2. One of the major food-borne pathogenic bacteria is *Listeria* spp. which is often found in food products. Hence, d-LAMP of *Salmonella* spp. and *Campylobacter* spp. may not be sufficient for surveillance test.

***Response*:** Thank you for your critical reading throughout our manuscript. We agreed with the reviewer's comments that *Listeria* spp. is one cause of the seriously severe foodborne diseases and it is a significant public health concern. However, according to the World Health Organization (WHO), *Campylobacter* and *Salmonella* are among the key global causes of diarrheal diseases (others including Norovirus and *E. coli*) (17). Both pathogens are highly prevalent in chicken meat, which is considered as the main food vehicle of infection for human (18-20). The control of these pathogens in chicken meat is also essential for avoiding sanitary barriers and reducing contamination, which is very useful for the exporter of this product (21). Thus, our d-LAMP-LFB assay developed in this work could be used for the simultaneous detection of *Campylobacter* and *Salmonella* with applications in food products.

3. The d-LAMP condition is arbitrary for detection of both *Salmonella* spp. and *Campylobacter* spp.as well as the selected gene for primer design. Hence, this may affect the sensitivity or limit of detection of each bacterial test. Generally, the LOD of d-LAMP is less sensitive than a single target detection.

***Response*:** We appreciate your comment. So far, the normal molecular detection technology coupled with enrichment procedure was limited to detect only one target template either *Campylobacter* or *Salmonella* spp. in food (22, 23), as it is challenging to detect both pathogens simultaneously, due to the fact that their growth requirements on different selective broths as well as slower growth of *Campylobacter* under microaerobic conditions (24). In this study, we successfully detected both pathogens in only a single reaction. We found that s- and d-LAMP-LFB results showed that there was no obvious difference in the detection limit for *Salmonella*. However, the limit of detection of d-LAMP was found to be less sensitive by ten-time for *Campylobacter*. Even though, the LOD of d-LAMP less sensitive than s-LAMP in this study, however, d-LAMP sensitivity was comparable to previous publications (22, 23, 25, 26), and commercially available real-time PCR kits such as BAX® *Campylobacter coli*/*jejuni*/*lari* assay (DuPont Qualicon), BAX® *Salmonella* assay (Hygiena), BIOTECON foodproof® *Campylobacter* Detection Kit (Biotecon Diagnostics) and BIOTECON foodproof® *Salmonella* Detection Kit (Biotecon Diagnostics). Thus, our developed d-LAMP-LFB assay has the potential to produce considerable savings of time and found to be superior to other previous studies for detecting of both pathogens simultaneously.

Are there any reasons that the authors select 16S rRNA and *invB* for primer and probe design? What is/are the feature (s) or advantage (s) of these genes over the others?

***Response*:** As commented by the reviewer, according to food safety of international criteria, regulation standards have strict guidelines, stating that the analyzed sample must have no *Campylobacter* and *Salmonella* contamination (27-29). Thus, the genetic marker used for designing of LAMP primer must have conserve regions for all strains of either *Campylobacter* or *Salmonella*. In this study, our d-LAMP-LFB assay was developed for simultaneous detection of *Campylobacter* spp. and *Salmonella* spp., by targeting 16S rRNA (GenBank accession no. AL111168.1) and *invB* (GenBank accession no. CP009102.1) genes, respectively. The 16S rRNA gene, coding 16S ribosomal RNA, was highly conserved among *Campylobacter*, and it was present in the chromosomes of all *Campylobacter species*. It was the most frequently targeted gene for

designing primers (30). Meanwhile, the *Salmonella* invasion protein B (*invB*), has been shown to be virulence factors of *Salmonella* (31), and this gene has been demonstrated to exist and unique within *Salmonella* strains but not in non-*Salmonella* (32). This evidence indicates that 16S rRNA and *invB* genes could serve as the reliable and accurate target genes for detection of all *Campylobacter* spp. and *Salmonella* spp., respectively. In addition, the specificity of our d-LAMP-LFB assay was successful investigated, and the positive results were generated in the assay of *Campylobacter* and *Salmonella* strains but not for non-*Campylobacter* and non-*Salmonella.* In conclusion, the advantage by using these target genes could be a valuable tool for detecting both target pathogens simultaneously in a variety of samples. We also added more information of these target genes in the methodology part on **Page 6, Line 122-123.**

4. The author should indicate the melting temperature of each primer in Table 1.

***Response*:** As suggested by the reviewer, LAMP primer designing by Primer Explorer V5 software program (http://primerexplorer.jp/lampv5e/index.html) provided Tm value of four LAMP primers as shown in Fig 1 below. However, the forward inner primer (FIP), which consist of the F2 and F1c primers and backward inner primer (BIP), which consists of B2 and B1c primers were linked with a poly T linker "TTTT" between F1c - F2 and B1c - B2. The actual Tm value of the FIP and BIP primers were not estimated by a default setting. In addition, all of scientific articles did not include Tm value of LAMP primer in the article. Thus, we think that the melting temperature (Tm) of each primer was not necessary for the experimental design. Therefore, this information was omitted in Table 1.

Primer Information Save

1 ID:3 dimer(minimum)dG=-1.60

label	5'pos	3'pos	len	Tm	5'dG	3'dG	GCrate	Sequence
F3	422	441	20	59.20	-4.92	-4.41	0.50	CGATCTGCTGGAACTCAACT
B3	624	641	18	60.53	-5.29	-4.66	0.61	CATGCTCCACCGCTTGTG
FIP			38					TAGGGCGTGGACTACCAGGG-GACGCTAAGGCGCGAAAG
BIP			42					ACGCATTAAGTGTACCGCCTGG-GGTCCCCGTCTATTCCTTTG
F2	442	459	18	60.45	-6.42	-4.79	0.61	GACGCTAAGGCGCGAAAG
F1c	485	504	20	64.85	-5.77	-6.29	0.65	TAGGGCGTGGACTACCAGGG
B2	602	621	20	59.34	-6.30	-4.61	0.55	GGTCCCCGTCTATTCCTTTG
B1c	555	576	22	64.83	-6.12	-6.69	0.55	ACGCATTAAGTGTACCGCCTGG

Primer Information Save

1 ID:8 dimer(minimum)dG=-2.31

label	5'pos	3'pos	len	Tm	5'dG	3'dG	GCrate	Sequence
F3	89	108	20	60.32	-5.30	-5.19	0.55	CGGAAAAGAAGCGACAGAGG
B3	262	280	19	59.74	-8.30	-4.29	0.53	CGGGCGACATTTGACAGAT
FIP			39					GGCTTTCGCTTTGGCGTCTG-GCCTTAGATAAGGCCACGG
BIP			40					TGACCAAATTCCAGGGAACGGC-CTGCTCACCCTGGGAAAC
F2	117	135	19	59.28	-4.93	-6.78	0.58	GCCTTAGATAAGGCCACGG
F1c	157	176	20	64.16	-5.35	-5.68	0.60	GGCTTTCGCTTTGGCGTCTG
B2	240	257	18	59.25	-5.59	-4.62	0.61	CTGCTCACCCTGGGAAAC
B1c	196	217	22	65.21	-5.42	-6.68	0.55	TGACCAAATTCCAGGGAACGGC

Fig 1. The detail of primer information window generated by Primer Explorer V5 software program designed on the target sequence of 16S rRNA (upper) and *invB* genes (lower).

Reviewer #2: Specific comments:

Discussion:

1. **Line 415-419; previous version:** are repeated to each other. This should be re-written. In my opinion detection without pre-enrichment is much more challenge.

Response**:** Thank you for your critical reading throughout our manuscript. We have rewritten the discussion part according to the reviewer's concerns on **Page 19, Line 417-421** The point that was concerned by the reviewer is that "detection without pre-enrichment is much more challenge". The control of these two pathogens is essential for food safety. Detection of both organisms cannot be confirmed by the molecular techniques without an enrichment procedure (33). Thus, an enrichment process should be included in our developed LAMP-LFB assay in this study.

2. **Line 422-425; previous version:** The authors should point out that the less LOD of *Campylobacter* spp. is the limitation of d-LAMP when compared to the single detection. Hence, the primers should be re-designed or changed the gene target,

***Response*:** We appreciate your comment. 16S rRNA and *invB* genes for designing LAMP primers in this study were examined as 100% specificity for *Campylobacter* and *Salmonella*, respectively. The positive results were generated in the assay of only *Campylobacter* and *Salmonella* strains, but not for non-*Campylobacter* and non-*Salmonella*. We think that these target genes are already suitable for detection of both pathogens. However, we agreed with the reviewer's comment that successful amplification of the d-LAMP relies on the designed primers. The primer sets should have similar melting temperature (Tm) and genomic GC content to produce a successful d-LAMP assay. In this study, two set of LAMP primers for *Campylobacter* and *Salmonella* were critically examined, which showed similar Tm and GC content. However, amplification efficiency of 16S rRNA *Campylobacter* might be lower than that of *invB Salmonella*. The interaction may occur between the multiple primer set in d-LAMP that results in primer dimers or secondary structures. Thus, the condition of d-LAMP is necessary to be further optimized by adding some chemicals such as methyl sulfoxide (DMSO) could elevate amplification efficiencies by inhibiting secondary structures in the DNA primers (13, 34). We have therefore added information in the discussion part on **Page 20, Line 428-432.**

3. **Line 445-451; previous version:** the discussion of two false positive samples are conflict to the purposes of this study. Since the samples were pre-enriched prior to d-LAMP, the contamination of dead cells is not quite possible. The second reason as the author discussed that d-LAMP-LFD could detect viable but non-culturable in XLD agar indicated that the specificity of the test is questionable. So, this is another the limitation of the test.

***Response*:** Thanks for your careful reading of our manuscript and for giving such constructive comments which helped improving the quality of the paper. The first comment by the reviewer is that "the contamination of dead cells is not quite possible".

***Response*:** Molecular techniques such as PCR, real-time PCR and LAMP were nucleic acid amplification-based techniques, which were considered with high sensitivity (1, 3). These techniques possibly detected both viable and non-viable bacteria contained in the samples (35). The high concentration of non-viable cells existence in the initial samples can be detected by those

molecular techniques (9), even though food samples were enriched to dilute the number of non-viable cell and increase the ability to detect viable cell prior testing by those molecular assays (9, 35-37). Thus, this is the limitations of those nucleic acid based assays in the detection of pathogens from foods, especially in extremely sensitive detection assay like LAMP (4). Thus, an interpretation of test results by those molecular assays must be carefully considered. The addition of DNase prior DNA extraction step (38) may resolve the probability of false positives. In addition, increasing of the time for an enrichment culture step for standard culture method can be excluded the truly false-positive LAMP results for ensuring that these samples were both truly positive for LAMP and standard culture method.

The second comment by the reviewer is that "d-LAMP-LFD could detect viable but non-culturable in XLD agar indicated that the specificity of the test is questionable. So, this is another the limitation of the test".

***Response*:** The viable but non-culturable bacterial cells presented in foods are also detected by molecular techniques (35). It can possibly cause false positive LAMP results by detecting of sub-lethally damaged bacterial cells (still viable) in the samples (9), which may be non-culturable on the XLD agar or under the culture conditions used in this study. This sample cannot thus be detected by the culture-based method, and it could be cultured in other selective agar of choice like Hektoen Enteric agar (HE) or Brilliance *Salmonella* agar (BE) according to standardized microbiological procedures (16, 39) as shown in the discussion part as well as xylose–lysine–tergitol (Niaproof) 4 agar (XLT4) and Miller-Mallinson agar (MM), which can enhance the specificity and sensitivity of *Salmonella* detection and the reduction of competing Enterobacteriaceae other than *Salmonella*. XLT4 also detected more *Salmonella*-positive samples of poultry origin by increasing recovery rates than did HE and XLD plates (40). These evidences indicated that using other selective agar is advantage over commonly used (XLD) recommended by the standard method for identification of *Salmonella* (16, 39), which might cause false positive LAMP testing. In conclusion, it is unclear whether these are truly false-positive results. The discrepancies between LAMP-LFB and culture results may also be attributed to differential-selective agar and culture conditions for isolating certain *Salmonella* serotypes (9). Test results must be considered for interpretation.

4. **Line 459-463; previous version:** The authors mentioned the improvement of sensitivity of *Campylobacter* spp. using d-LAMP-LFD assay by extending incubation time or change culture medium. This confirmed that the detection of *Campylobacter* spp. was not rapid and simple as indicated by the authors.

Response**:** We appreciate your comment. We proposed the take home massage that the one false negative reported in this study was probably due to *Campylobacter* cell density, being below the minimum detection limit of d-LAMP-LFB. An enrichment could circumvent this problem by bringing *Campylobacter* cell numbers into the detection range of d-LAMP-LFB assay. However, *Campylobacter* is fastidious and slow-growing organisms in enrich culture. They required enrichment time for at least approximately 48 h under microaerobic conditions (21, 41), while our enrichment time for 24 h is enough for detecting *Campylobacter*, which was comparable to other previous studies and commercially available real-time PCR kits. We have therefore rewritten this point in the discussion part on **Page 21, Line 466-468** and **Page 22, line 469**.

5. **Line 480-485; previous version:** The discussion is unacceptable due to the d-LAMP LFD assay has some limitations as followed.

a. The assay required pre-enrichment step which is time-consuming and tedious.

Response**:** As commented by the Reviewer, our developed d-LAMP-LFB assay coupled with enrichment procedure can be used for detecting both *Campylobacter* and *Salmonella* in foods simultaneously, which provides a test result within two 8-h working shifts, more time-efficient and labor saving than that of the gold standard culture-based method. Even though the molecular technique is a highly sensitive detection method, but it is not of value if applied directly for the detection of pathogens in foods (6). An enrichment step is still required for detecting pathogens in foods not only enhance the cell number prior to testing by molecular detection method but also inhibits the growth of background flora and dilutes inhibitor present in different food matrices, which can interfere amplification reaction (8). Without requiring enrichment step prior molecular testing, false-negative testing may occur, which increases the risk of foodborne illness in human due to contaminated food. As well, false-negative test results could be of critical importance to food safety regulation whose samples are compromised when positive sample is misinterpreted as negative. Thus, the inclusion of a minimum enrichment time for at least 24 h is necessary in a detection protocol in this study.

b. The LOD of the assay is less sensitive than a single detection especially for *Campylobacter* spp.

***Response*:** As commented by the Reviewer, the LOD for *Campylobacter* of our developed d-LAMP-LFB assay is less sensitive than that of a single detection. However, the detection range of LOD of our assay is comparable to a previous study (22) and commercially available real-time PCR rapid detection kits. In addition, d-LAMP-LFB assay here can potentially facilitate simultaneous detection and correctly distinguish *Campylobacter* and *Salmonella* in only a single reaction, which is considered more rapid and convenient than a single testing. Therefore, developed d-LAMP-LFB assay could be an alternative method to rapidly detect both pathogens in foods.

c. The specificity of the assay is questionable since the viable non-culturable in XLD agar could interfere the results.

***Response*:** Thank you for pointing this out. Two false positive LAMP results were interpreted for *Salmonella* detection. We have already discussed that these false positive results may probably due to detect dead cells and/or viable-non culturable cells in the discussion part. We also drew the schematic representation of the standard culture method compared with LAMP-LFB assay in Fig 2 below. We think that our developed d-LAMP-LFB detected false positive results due to the existence of sub-lethally damaged *Salmonella* cells (still viable) in the samples, which may be non-culturable (9) on the medium (XLD) used in this study. It may by cultured on differential-selective agar and culture conditions for isolating certain *Salmonella* (9) serotypes such as Hektoen Enteric agar (HE), Brilliance *Salmonella* agar (BE), xylose–lysine–tergitol (Niaproof) 4 agar (XLT4) and Miller-Mallinson agar (MM). Two false positive LAMP results in this study may not be considered as a truly-false positive result. Thus, the result interpretation could explain the limited detection specificity of molecular method, especially when false positive result was present in testing

6. In conclusion of my comment, this confirmed that the d-LAMP-LFD for detection of *Salmonella* spp. and *Campylobacter* spp. was not rapid and simple as indicated by the authors. In contrary, it is quite time-consuming and tedious since it still depends on pre-enrichment process.

***Response*:** Thank you very much for your comment. Because of the increasing importance of *Salmonella* spp. and *Campylobacter* spp., rapid and simple methods for their detection are required. Even though the culture-based method is still used as the gold standard for detection of these pathogens, the routine procedures required multiple subculture steps, isolation, confirmation and species identification, which required about 7 days for a conclusive negative result and 7 to 14 days for confirmation of a positive finding (15, 16). They have their own limitation including time-consuming and labor intensive (1, 3). Our developed d-LAMP-LFB assay in contrast allow rapid detection of both pathogens simultaneously within two 8-h working shifts, more time-efficient and labor saving. An additional of enrichment step in procedure enables molecular detection assays to efficiently and accurately detected low numbers of pathogens in food samples, so this step is still essential for nucleic acid based assays to reduce the chance of false negatives result (7-12). In addition, our d-LAMP-LFB assay is also a cost-effective and easy to perform under isothermal condition using just only simple water bath, eliminating the use of highly sophisticated instrument. Thus, our developed d-LAMP-LFB assay serves as an alternative tool to standard culture method with the advantage of less time consuming in detecting both pathogens simultaneously.

References

1. Law JW, Ab Mutalib NS, Chan KG, Lee LH. Rapid methods for the detection of foodborne bacterial pathogens: principles, applications, advantages and limitations. Frontiers in microbiology. 2014;5:770.
2. Dhama K, Karthik K, Chakraborty S, Tiwari R, Kapoor S, Kumar A, et al. Loop-mediated isothermal amplification of DNA (LAMP): a new diagnostic tool lights the world of diagnosis of animal and human pathogens: a review. Pakistan journal of biological sciences : PJBS. 2014;17(2):151-66.
3. Zhao X, Lin CW, Wang J, Oh DH. Advances in rapid detection methods for foodborne pathogens. Journal of microbiology and biotechnology. 2014;24(3):297-312.
4. Wong Y-P, Othman S, Lau Y-L, Radu S, Chee H-Y. Loop-mediated isothermal amplification (LAMP): a versatile technique for detection of micro-organisms. J Appl Microbiol. 2018;124(3):626-43.

5. Myint MS, Johnson YJ, Tablante NL, Heckert RA. The effect of pre-enrichment protocol on the sensitivity and specificity of PCR for detection of naturally contaminated *Salmonella* in raw poultry compared to conventional culture. Food Microbiol. 2006;23(6):599-604.
6. Kaneko I, Miyamoto K, Mimura K, Yumine N, Utsunomiya H, Akimoto S, et al. Detection of enterotoxigenic *Clostridium perfringens* in meat samples by using molecular methods. Appl Environ Microbiol. 2011;77(21):7526-32.
7. Lim HS, Zheng Q, Miks-Krajnik M, Turner M, Yuk HG. Evaluation of commercial kit based on loop-mediated isothermal amplification for rapid detection of low levels of uninjured and injured *Salmonella* on duck meat, bean sprouts, and fishballs in Singapore. Journal of food protection. 2015;78(6):1203-7.
8. Cheng CM, Lin W, Van KT, Phan L, Tran NN, Farmer D. Rapid detection of *Salmonella* in foods using real-time PCR. Journal of food protection. 2008;71(12):2436-41.
9. Hong Y, Berrang ME, Liu T, Hofacre CL, Sanchez S, Wang L, et al. Rapid detection of *Campylobacter coli*, *C. jejuni*, and *Salmonella enterica* on poultry carcasses by using PCR-enzyme-linked immunosorbent assay. Appl Environ Microbiol. 2003;69(6):3492-9.
10. Mester P, Wagner M, Rossmanith P. Molecular Enrichment for Qualitative Molecular Pathogen Detection in Food. Food Analytical Methods. 2018;11(5):1251-6.
11. Yamazaki W, Taguchi M, Kawai T, Kawatsu K, Sakata J, Inoue K, et al. Comparison of loop-mediated isothermal amplification assay and conventional culture methods for detection of *Campylobacter jejuni* and *Campylobacter coli* in naturally contaminated chicken meat samples. Appl Environ Microbiol. 2009;75(6):1597-603.
12. Cremonesi P, Pisani LF, Lecchi C, Ceciliani F, Martino P, Bonastre AS, et al. Development of 23 individual TaqMan® real-time PCR assays for identifying common foodborne pathogens using a single set of amplification conditions. Food Microbiol. 2014;43:35-40.
13. Yang Q, Domesle KJ, Ge B. Loop-mediated isothermal amplification for *Salmonella* detection in food and feed: current applications andfFuture directions. Foodborne pathogens and disease. 2018;15(6):309-31.
14. Thung T, Lee E, Wai G, Pui C, Kuan C, Premarathne J, et al. A review of culture-dependent and molecular methods for detection of *Salmonella* in food safety. Food Research 2019;3(6):622-7.

15. ISO10272:2006(E). Microbiology of food and animal feeding stuffs-horizontal method for the detection and enumeration of *Campylobacter* spp., International Standard Organization.
16. ISO6579:2002(E). Microbiology of food and animal feeding stuffs-horizontal method for the detection of *Salmonella* spp., International Standard Organization.
17. WHO. Diarrhoeal diseases: WHO estimates of the global burden of foodborne diseases. 2015. Available from: https://www.who.int/foodsafety/areas_work/foodborne-diseases/infographics_combined_en.pdf.
18. WHO. *Salmonella* and *Campylobacter* in chicken meat, microbiological risk assessment series 19, FAO/WHO expert meeting report. [revised 2009; cited 2020 Aug 1]. Available from: https://www.who.int/foodsafety/publications/mra19/en/.
19. Thames HT, Theradiyil Sukumaran A. A review of *Salmonella* and *Campylobacter* in broiler meat: emerging challenges and food safety measures. Foods (Basel, Switzerland). 2020;9(6):776.
20. Rukambile E, Sintchenko V, Muscatello G, Kock R, Alders R. Infection, colonization and shedding of *Campylobacter* and *Salmonella* in animals and their contribution to human disease: a review. Zoonoses and public health. 2019;66(6):562-78.
21. Alves J, Marques VV, Pereira LFP, Hirooka EY, De Oliveira TCRM. Multiplex PCR for the detection of *Campylobacter* spp. and *Salmonella* spp. in chicken meat. Journal of Food Safety. 2012;32(3):345-50.
22. Thongphueak D, Chansiri K, Sriyapai T, Areekit S, Santiwatanakul S, Wangroongsarb P. Development of the rapid test kit for the identification of *Campylobacter* spp. based on loop-mediated isothermal amplification (LAMP) in combination with a lateral flow dipstick (LFD) and gold nano-DNA probe (AuNPs). Science & Technology Asia. 2019;24(1):63-71.
23. Liu Z, Zhang Q, Yang NN, Xu MG, Xu JF, Jing ML, et al. Rapid and sensitive detection of *Salmonella* in chickens using loop-mediated isothermal amplification combined with a lateral flow dipstick. Journal of microbiology and biotechnology. 2019;29(3):454-64.
24. Wolffs PF, Glencross K, Norling B, Griffiths MW. Simultaneous quantification of pathogenic *Campylobacter* and *Salmonella* in chicken rinse fluid by a flotation and real-time multiplex PCR procedure. International journal of food microbiology. 2007;117(1):50-4.

25. Yu J, Xing J, Zhan X, Yang Z, Qi J, Wei Y, et al. Improvement of loop-mediated isothermal amplification combined with chromatographic flow dipstick assay for *Salmonella* in food samples. Food Analytical Methods. 2020;13(7):1398-408.

26. Mei X, Zhai X, Lei C, Ye X, Kang Z, Wu X, et al. Development and application of a visual loop-mediated isothermal amplification combined with lateral flow dipstick (LAMP-LFD) method for rapid detection of *Salmonella* strains in food samples. Food Control. 2019;104:9-19.

27. FSANZ/Australia New Zealand. Food Standards Australia New Zealand, Compendium of microbiological criteria for food. [revised 2018 Sep ; cited 2020 Aug 1]. Available from: https://www.foodstandards.gov.au/publications/Documents/Compedium%20of%20Microbiological%20Criteria/Compendium_revised-jan-2018.pdf.

28. EC. Commission Regulation (EC) No 2073/ 2005 of 15 November 2005 on microbiological criteria for foodstuffs. [revised 2005 Nov 15 ; cited 2020 Aug 1]. Available from: https://eur-lex.europa.eu/legal-content/EN/TXT/PDF/?uri=CELEX:02005R2073-20140601&from=EN.

29. DLD/Thailand. Department of Livestock Development,Thailand. Microbiological guideline for chilled / frozen meat & poultry meat. [revised 2008; cited 2020 Aug 1]. Available from: http://qcontrol.dld.go.th/images/law/regulation/MicrobiologicalSTDforLivestockProducts.PDF.

30. Ricke SC, Feye KM, Chaney WE, Shi Z, Pavlidis H, Yang Y. Developments in rapid detection methods for the detection of foodborne *Campylobacter* in the United States. Frontiers in microbiology. 2018;9:3280.

31. Sirsat SA, Burkholder KM, Muthaiyan A, Dowd SE, Bhunia AK, Ricke SC. Effect of sublethal heat stress on *Salmonella* Typhimurium virulence. J Appl Microbiol. 2011;110(3):813-22.

32. Porter SB, Curtiss R, 3rd. Effect of *inv* mutations on *Salmonella* virulence and colonization in 1-day-old White Leghorn chicks. Avian diseases. 1997;41(1):45-57.

33. Alves J, Hirooka EY, de Oliveira TCRMJL-FS, Technology. Development of a multiplex real-time PCR assay with an internal amplification control for the detection of *Campylobacter* spp. and *Salmonella* spp. in chicken meat. LWT Food Sci Technol. 2016;72:175-81.

34. Shao Y, Zhu S, Jin C, Chen F. Development of multiplex loop-mediated isothermal amplification-RFLP (mLAMP-RFLP) to detect *Salmonella* spp. and *Shigella* spp. in milk. International journal of food microbiology. 2011;148(2):75-9.

35. Saiyudthong S, Phusri K, Buates S. Rapid detection of *Campylobacter jejuni*, *Campylobacter coli*, and *Campylobacter lari* in fresh chicken meat and by-products in Bangkok, Thailand, using modified multiplex PCR. Journal of food protection. 2015;78(7):1363-9.

36. Sridapan T, Tangkawsakul W, Janvilisri T, Kiatpathomchai W, Dangtip S, Ngamwongsatit N, et al. Rapid detection of *Clostridium perfringens* in food by loop-mediated isothermal amplification combined with a lateral flow biosensor. PloS one. 2021;16(1):e0245144.

37. Siala M, Barbana A, Smaoui S, Hachicha S, Marouane C, Kammoun S, et al. Screening and detecting *Salmonella* in different food matrices in southern Tunisia using a combined enrichment/real-time PCR method: correlation with conventional culture method. Frontiers in microbiology. 2017;8:2416.

38. Priya GB, Agrawal RK, Milton AAP, Mishra M, Mendiratta SK, Agarwal RK, et al. Development and evaluation of isothermal amplification assay for the rapid and sensitive detection of *Clostridium perfringens* from chevon. Anaerobe. 2018;54:178-87.

39. FDA-BAM. Bacteriological analytical manual chapter 5: *Salmonella*. Food & Drug Administration, Bacteriological analytical manual, U.S. Department of health and human services, Public health service, Washington DC. [revised 2020 Jul ; cited 2020 Aug 1]. Available from: https://www.fda.gov/food/laboratory-methods-food/bam-chapter-5-salmonella.

40. Mallinson ET, Miller RG, de Rezende CE, Ferris KE, deGraft-Hanson J, Joseph SW. Improved plating media for the detection of *Salmonella* species with typical and atypical hydrogen sulfide production. Journal of veterinary diagnostic investigation : official publication of the American Association of Veterinary Laboratory Diagnosticians, Inc. 2000;12(1):83-7.

41. Sails AD, Bolton FJ, Fox AJ, Wareing DR, Greenway DL. Detection of *Campylobacter jejuni* and *Campylobacter coli* in environmental waters by PCR enzyme-linked immunosorbent assay. Appl Environ Microbiol. 2002;68(3):1319-24.
