# Supplementary material for: Rapid and simultaneous detection of Campylobacter spp. and Salmonella spp. in chicken samples by duplex loop-mediated isothermal amplification coupled with a lateral flow biosensor assay
Source: PLoS One. 2021 Jul 1;16(7):e0254029. doi: 10.1371/journal.pone.0254029 (PMC8248736; doi:10.1371/journal.pone.0254029)
Supplement: Attachment — Submitted filename: Response to the reviewer 110621.pdf [file pone.0254029.s013.pdf]

Response to the reviewer' comments

Page numbers and lines mentioned refers to that of the main text of the revised manuscript with inline markup version.

Journal Requirements#:

1: Please review your reference list to ensure that it is complete and correct. If you have cited papers that have been retracted, please include the rationale for doing so in the manuscript text, or remove these references and replace them with relevant current references. Any changes to the reference list should be mentioned in the rebuttal letter that accompanies your revised manuscript. If you need to cite a retracted article, indicate the article's retracted status in the References list and also include a citation and full reference for the retraction notice.

Response**:** Thanks for your careful reading of our manuscript and for giving such constructive comments which helped improving the quality of the paper. The reference number 3, 6, 7, 8, 26, 27, 33, 36, 39, and 44 have been reviewed and replaced with relevant current references to ensure that the citation is complete and accurate.

Reviewer #3: General comments:

The manuscript PONE-D-21-06858R1, reports the development and application of the d-LAMP-LFB for the multiplex detection of two foodborne pathogens simultaneously. The article is well written and presents a great deal of work for the assessment of the important possibility to detect the presence of several food-borne pathogens in only one reaction tube and test. The authors claimed that is the first multiplex LAMP-LFB for detection of *Campylobacter* spp. and *Salmonella* spp.

Response**:** We thank the reviewer#3 for careful reading of our manuscript. We are grateful for your consideration of this manuscript.

Reviewer #3: Major comments:

There is a constant misunderstand between limit of detection (LOD) of an assay and the initial spiking/initial inoculum of bacterial cells. LOD is defined as the minimum detectable amount/concentration of the target (bacteria cells or DNA) on the sample prior to application in the tests. The initial inoculum is the initial bacterial concentration on sample before the enrichment in a selective medium. Authors should not interpret that low levels of initial inoculation used for the enrichment step are the LOD of the test. Therefore, we recommend that authors revise and explain better when they discuss and compare the LOD.

Response**:** Thank you for your critical reading throughout our manuscript. We agreed with the reviewer's comments that limit of detection (LOD) is important performance characteristics in method validation. Analytical sensitivity or LOD are used to describe the lowest concentration of an analyte that can be measured by an analytical procedure. In this study, the analytical sensitivity was used to assess the analysis of the lowest concentration of serially diluted genomic DNA templates as the "**sensitivity limit**". Meanwhile, in spiking study, the last dilution in each spiked sample that tested positive was defined as "**the lowest inoculated detection limit**" of d-LAMP-LFB assay for visualization of the LAMP products. This term has also been used in previous publications to determine the analytical sensitivity in spiking study (Techathuvanan et al., 2010). We therefore have rewritten these terms on **Page 2, Line 32; Page 6, Line 121; Page 9, Line 194; Page 11, Line 236, 238, 239 and 242; Page 16, Line 338, 339, 340, 341, 343, 348, 349, 350, 354, 356 and 357; Page 17, Line 358, 360, 361, 362, 365 and 367; Page 18, Line 398; Page 20, Line 433, 440, 441 and 441; Page 22, Line 481 and 483.**

Reference: Techathuvanan C, Draughon FA, D'Souza DH. Loop-mediated isothermal amplification (LAMP) for the rapid and sensitive detection of *Salmonella* Typhimurium from pork. Journal of food science. 2010;75(3):M165-72.

Reviewer #3: Specific comments:

Introduction:

1. Line 54-55; previous version: Authors would emphasize the importance of their work by adding in the introduction few sentences with information on the main bacteria causing diarrheal

diseases and the recommended control rules and not just mention the two studied bacteria and being so vague as mentioning “so the regulation standards have strict guidelines”.

***Response*:** We appreciate your comment. We revised the manuscript accordingly, please kindly see **Page 3, Line 48-58.**

2. Line 60; previous version: The article reports about a test to detect the presence of bacteria therefore it is not a diagnostic test.

***Response*:** We appreciate your comment. The phrase “establishing the laboratory diagnostic of these pathogens” has been rephrased to “detecting these pathogens present in food” on **Page 3, Line 64-65.**

3. Line 65; previous version: The authors can increase the value of their work by mentioning the other disadvantages of PCR in order to emphasize the advantage to use d-LAMP-LFB.

***Response*:** We appreciate your comment which helped improving the quality of the paper. To emphasize the advantage to use d-LAMP-LFB, we added more information of the advantage to use d-LAMP-LFB on **Page 4, Line 87-89.**

Methodology:

1. Line 103; previous version: Please, inform the source of the BHI broth applied.

***Response*:** Thank you for this remark. The phrase “Himedia, India” has been added on **Page 5, Line 110.**

2. Line 228; previous version: Please note that “the last dilution in each spiked sample that test positive” is not the detection limit (LOD) of the test but the inoculation level. This is a major concern with many other papers discussing the LOD. Anytime there is enrichment, the initial spiking level becomes irrelevant for determining the LOD of the test. What is more important is how many cells are going into the sample used in your LAMP assay. Nevertheless, it is important to mention the initial spiking level but it is wrong to use it as the LOD.

***Response*:** We appreciate your comment. We agreed with the reviewer’s comments. The LOD used in this study was the initial spiking level or lowest inoculated detection limits. Thus, the LOD in this study has been rephrased as lowest inoculated detection limits on **Page 11, Line 236**.

Result:

1. Line 352-355; previous version: Authors are reporting in this paragraph the initial spiking level that is the sample analytical LOD but not the LOD of the LAMP-LFB.

Response**:** As commented by the reviewer, the LOD of the LAMP-LFB was rephrased as lowest inoculated detection limits on **Page 16, Line 354-362.**

2. Line 355; Figure 5; previous version: Please, report as initial spiking level or sample analytical LOD.

Response**:** As suggested by the reviewer, the LOD of the LAMP-LFB was rephrased as lowest inoculated detection limits on **Page 17, Line 365 and 367.**

Discussion:

1. Line 432-445; previous version: I don't think that is the appropriate manner to discuss the results obtained since the authors did not determine how many CFU/ml they had in their enriched sample before DNA extraction and application on the LAMP-LFB. In addition, by claiming 0.04 CFU/g the authors are considering that they initiated the experiments by inoculating only one bacterium. True comparison of tests should also evaluate whole assay time. As the majority of tests and regulation standards seek to detect a single bacterium in 25 g of sample, the spiking level is irrelevant for comparison.

Response**:** Thank you for your critical reading throughout our manuscript. We have rewritten the discussion part on **Page 20, Line 441-458,** according to the reviewer's concerns on comparing the lowest inoculated detection limits and the time for enrichment procedure of our d-LAMP-LFB assay with other previous studies. The parenthesis indicated the lowest inoculated detection limits of the original report of other previous studies.

In addition, we apologize for such confusion at discussion part on page 21, line 465-467. This sentence is a redundancy sentence, and it has the same meaning and already describe on discussion part on page 22, line 487-499. Thus, these sentences on page 21, line 465-467 have been removed from the manuscript.
