# Supplementary material for: Rapid and simultaneous detection of Campylobacter spp. and Salmonella spp. in chicken samples by duplex loop-mediated isothermal amplification coupled with a lateral flow biosensor assay
Source: PLoS One. 2021 Jul 1;16(7):e0254029. doi: 10.1371/journal.pone.0254029 (PMC8248736; doi:10.1371/journal.pone.0254029)
Supplement: Attachment — Submitted filename: Comments for Manuscript Number PONE.docx [file pone.0254029.s010.docx]

Comments for Manuscript Number PONE-D-21-06858

1. The authors indicated that the d-LAMP is rapid and simple for simultaneous detection of *Salmonella* spp. and *Campylobacter* spp. However, this technique must rely on pre-enrichment which is the bottle neck of the over all process and make it not rapid and simple as claimed by the authors. Though the pre-enrichment has the major advantage in discrimination between dead and alive pathogenic bacteria, but it is tedious and time- consuming. Hence, the overall process starting from sample treatment could take approximately 24 hours or more which is not rapid and simple. As such, line 398-391 should be deleted or re-discussed since the d-LAMP combined with pre-enrichment could not be applied for field investigation.
2. One of the major food-borne pathogenic bacteria is *Listeria* spp. which is often found in food products. Hence, d-LAMP of *Salmonella* spp. and *Campylobacter* spp. may not be sufficient for surveillance test.
3. The d-LAMP condition is arbitrary for detection of both *Salmonella* spp. and *Campylobacter* spp.as well as the selected gene for primer design. Hence, this may affect the sensitivity or limit of detection of each bacterial test. Generally, the LOD of d-LAMP is less sensitive than a single target detection.

Are there any reasons that the authors select *16 SRNA* and *invB* for primer and probe design? What is/are the feature (s) or advantage (s) of these genes over the others?

4. The author should indicate the melting temperature of each primer in Table 1.
5. Lines 415-419 are repeated to each other. This should be re-written. In my opinion detection without pre-enrichment is much more challenge.
6. Lines 422-425, the authors should point out that the less LOD of *Campylobacter* spp. is the limitation of d-LAMP when compared to the single detection. Hence, the primers should be re-designed or changed the gene target,
7. Lines 445-451, the discussion of two false positive samples are conflict to the purposes of this study. Since the samples were pre-enriched prior to d-LAMP, the contamination of dead cells is not quite possible. The second reason as the author discussed that d-LAMP-LFD could detect viable but non-culturable in XLD agar indicated that the specificity of the test is questionable. So, this is another the limitation of the test.
8. Lines 459-463, The authors mentioned the improvement of sensitivity of *Campylobacter* spp. using d-LAMP-LFD assay by extending incubation time or change culture medium. This confirmed that the detection of *Campylobacter* spp. was not rapid and simple as indicated by the authors.
9. Lines 480-485, The discussion is unacceptable due to the d-LAMP LFD assay has some limitations as followed.
   a. The assay required pre-enrichment step which is time-consuming and tedious.
   b. The LOD of the assay is less sensitive than a single detection especially for *Campylobacter* spp.
   c. The specificity of the assay is questionable since the viable non-culturable in XLD agar could interfere the results.
10. In conclusion of my comment, this confirmed that the d-LAMP-LFD for detection of *Salmonella* spp. and *Campylobacter* spp. was not rapid and simple as indicated by the authors. In contrary, it is quite time-consuming and tedious since it still depend on pre-enrichment process.
