# Supplementary material for: Rapid and simultaneous detection of Campylobacter spp. and Salmonella spp. in chicken samples by duplex loop-mediated isothermal amplification coupled with a lateral flow biosensor assay
Source: PLoS One. 2021 Jul 1;16(7):e0254029. doi: 10.1371/journal.pone.0254029 (PMC8248736; doi:10.1371/journal.pone.0254029)
Supplement: Attachment — Submitted filename: renamed_f2f35.docx [file pone.0254029.s012.docx]

The manuscript PONE-D-21-06858R1, reports the development and application of the d-LAMP-LFB for the multiplex detection of two foodborne pathogens simultaneously. The article is well written and presents a great deal of work for the assessment of the important possibility to detect the presence of several food-borne pathogens in only one reaction tube and test. The authors claimed that is the first multiplex LAMP-LFB for detection of *Campylobacter* spp and *Salmonella* spp.

Major comments

There is a constant misunderstand between limit of detection (LOD) of an assay and the initial spiking/initial inoculum of bacterial cells. LOD is defined as the minimum detectable amount/concentration of the target (bacteria cells or DNA) on the sample prior to application in the tests. The initial inoculum is the initial bacterial concentration on sample before the enrichment in a selective medium. Authors should not interpret that low levels of initial inoculation used for the enrichment step are the LOD of the test. Therefore, we recommend that authors revise and explain better when they discuss and compare the LOD.

Please, see the specific comments below.

Line 228 – Please note that “the last dilution in each spiked sample that test positive” is not the detection limit (LOD) of the test but the inoculation level. This is a major concern with many other papers discussing the LOD. Anytime there is enrichment, the initial spiking level becomes irrelevant for determining the LOD of the test. What is more important is how many cells are going into the sample used in your LAMP assay. Nevertheless, it is important to mention the initial spiking level but it is wrong to use it as the LOD.

Lines 352-355 – Authors are reporting in this paragraph the initial spiking level that is the sample analytical LOD but not the LOD of the LAMP-LFB.

Figure 5 – Please, report as initial spiking level or sample analytical LOD.

Lines 432- 445 – I don’t think that is the appropriate manner to discuss the results obtained since the authors did not determined how many CFU/ml they had in their enriched sample before DNA extraction and application on the LAMP-LFB. In addition, by claiming 0.04 CFU/g the authors are considering that they initiated the experiments by inoculating only one bacterium. True comparison of tests should also evaluate whole assay time. As the majority of tests and regulation standards seek to detect a single bacterium in 25g of sample, the spiking level is irrelevant for comparison.

Minor comments

Lines 54-55 – Authors would emphasize the importance of their work by adding in the introduction few sentences with information on the main bacteria causing diarrheal diseases and the recommended control rules and not just mention the two studied bacteria and being so vague as mentioning “so the regulation standards have strict guidelines”.

Line 60 – The article reports about a test to detect the presence of bacteria therefore it is not a diagnostic test.

Line 65 – The authors can increase the value of their work by mentioning the other disadvantages of PCR in order to emphasize the advantage to use d-LAMP-LFB.

Line 103 – Please, inform the source of the BHI broth applied.
